# Antioxidant Senotherapy by Natural Compounds: A Beneficial Partner in Cancer Treatment

**DOI:** 10.3390/antiox14020199

**Published:** 2025-02-10

**Authors:** Yulia Aleksandrova, Margarita Neganova

**Affiliations:** Nesmeyanov Institute of Organoelement Compounds, Russian Academy of Sciences, Vavilova St. 28, Bld. 1, Moscow 119991, Russia; aleksyu@ineos.ac.ru

**Keywords:** senescent cells, cellular senescence, senescence-associated secretory phenotype, senotherapy, senolytic agents, antioxidant drugs, natural products

## Abstract

Aging is a general biological process inherent in all living organisms. It is characterized by progressive cellular dysfunction. For many years, aging has been widely recognized as a highly effective mechanism for suppressing the progression of malignant neoplasms. However, in recent years, increasing evidence suggests a “double-edged” role of aging in cancer development. According to these data, aging is not only a tumor suppressor that leads to cell cycle arrest in neoplastic cells, but also a cancer promoter that ensures a chronic proinflammatory and immunosuppressive microenvironment. In this regard, in our review, we discuss recent data on the destructive role of senescent cells in the pathogenesis of cancer. We also identify for the first time correlations between the modulation of the senescence-associated secretory phenotype and the antitumor effects of naturally occurring molecules.

## 1. Introduction

The definition of cellular senescence has undergone significant changes since Hayflick and Morehead first made the groundbreaking discovery in the 1960s. They observed replicative senescence in human fibroblasts and noted that fibroblasts reach a limit in their ability to proliferate, having undergone a maximum number of divisions [1]. To summarize the many formulations of the term “cellular senescence” that exist today, this phenomenon can be defined as “a heterogeneous state of cells, expressed as a stable exit from the cell cycle and subsequent radical morphological and physiological changes (in particular, aberrant metabolism [2], DNA damage [3], violation in the secretion of proinflammatory cytokines, chemokines and growth factors [4,5], etc.), which arrest further proliferative potential of the cells” [6].

Although we tend to think of senescent cells as accumulating with age, disrupting normal tissue physiology and forming foci of progressive functional decline, in fact cells can also undergo accelerated senescence. Such premature senescence is caused by a wide range of stress stimuli (termed “stress-induced premature senescence (SIPS)”), such as oxidative stress [7,8], metabolic dysfunction [9], exposure to pathogenic bacteria or viruses [10], oncogenic stress [11], or therapy-induced stress [12,13]. Similar to physiological aging, the induction of senescence in response to these stress stimuli results in cell cycle arrest. It for many years was considered solely as a protective mechanism against disease progression, particularly cancer. This paved the way for the concept of creating a “prosenescence” therapeutic strategy for cancer that induces the formation of senescent cells [14], termed “therapy-induced senescence” (TIS). In particular, this approach was in high demand at the beginning of the 21st century. A large number of research groups published review papers and experimental studies emphasizing the role of senescence as a “powerful weapon” for cancer eradication [14,15,16]. Moreover, it became known that the key effector mechanism of a large number of clinically approved anticancer drugs is precisely the induction of senescent cells. Namely, tamoxifen, sold under the brand name “Nolvadex” and the gold standard in the treatment of estrogen receptor-positive breast cancer, has a chronic damaging effect on tissues through the formation of the senescence-associated secretory phenotype (SASP) [17,18]. Oncogene-induced senescence is also a tumor suppression mechanism for the platinum coordination complex cisplatin [19,20], used as a chemotherapeutic drug for the treatment of a wide range of malignant neoplasms, in particular, brain tumors, genitourinary cancer, gastrointestinal cancer [21], etc.

However, a few years later, the hypothesis of the exclusive benefit of senescent cells in antitumor protection, consisting in stopping the development and progression of cancer, was replaced by an understanding of the dual role of aging in tumor cells [22]. It became obvious that the TIS strategy can only serve as an initial stage of antitumor therapy, while a promising direction at present is the combination of “prosenescence” therapy with senotherapy aimed at the eradication of senescent cells.

The truly recently formulated term “senotherapy” describes the process of rejuvenation of the organism by using pharmacological agents that implement their action through senolysis (selective eradication of senescent cells) or senomorphic intervention (reversal of disorders caused by cellular senescence) [23,24,25].

Although the vast majority of published studies describe the prospects of senotherapeutic strategies on non-tumor cells (mainly for the treatment of age-related diseases [26,27,28,29], as well as for prolonging lifespan and achieving healthy longevity [30]), there is a certain pool of data that indisputably proves the adverse effects of TIS, arising as a result of the treatment of malignant neoplasms. As mentioned above, in recent years, a critical mass of evidence has accumulated that such a promising TIS strategy can play a pro-tumorigenic role. So, TIS can promote tumor relapse and its aggressive growth [31,32] by returning senescent cells to the cell cycle and, as a result, resuming the proliferative potential. Moreover, the accumulation of senescent cells can explain the presence of side effects caused by the state of a permanent proinflammatory and immunosuppressive microenvironment [33]. In other words, in addition to the beneficial effects of this phenomenon, the accumulation of senescent tumor cells may contribute to adverse outcomes of traditional cancer therapy, including the emergence of a more malignant phenotype.

In this review, in addition to generalizing and clearly structuring the currently known data on cellular senescence, we present an extrapolation of this information in the context of the role of senescent cells in the pathogenesis of malignant neoplasms. Moreover, when describing the senotherapeutic potential of molecules, our work for the first time investigated the relationship between the effect of natural products on SASP and therapeutic effects associated mainly with their antioxidant potential in the cancer therapy context. It is expected that the approach we used may allow specialists in the field of medicinal chemistry to take a new look at the prospects of using the presented molecules in terms of senolytic antitumor drugs.

## 2. The Role of the Senescence-Associated Secretory Phenotype (SASP) in Cancer Pathogenesis

As mentioned above, senescent cells continuously produce factors that form the senescence-associated secretory phenotype, also known as the senescence-messaging secretome (SMS). The definition of SASP can be formulated as “a condition characterized by the permanent secretion of proteases, chemokines, cytokines, and growth factors by senescent cells into the tissue microenvironment in response to extracellular or intracellular stimuli” [34]. It is believed that this phenotype regulates cell behavior and determines tissue function in various pathologies.

In general, all factors produced by SASP can be divided into two groups: (1) soluble signaling molecules (proteases, growth factors, cytokines, and chemokines, as well as non-protein factors); (2) insoluble factors. Table 1 shows the data on the secretion features of the most common specific representatives of each group in senescent cells.

Based on Table 1, it is easy to see that in most cases, senescence is accompanied by an increase in the expression of signaling factors. This leads to non-autonomous transmission of damaging signals from senescent cells to neighboring cells of normal origin, causing damage to them.

The process of SASP formation and senescence in general is influenced by a variety of factors that form signaling pathways; of these, a critical role is played by the nuclear factor NF-kappaB pathway (NF-kB) [79], the phosphatidylinositol 3-kinase—protein kinase AKT—mammalian target of rapamycin (PI3K-AKT-mTOR) [80], the p38 MAPK (mitogen-activated protein kinase) pathway [80], and CCAAT enhancer binding protein beta (C/EBP-β) [81] activation.

**Figure 1:**
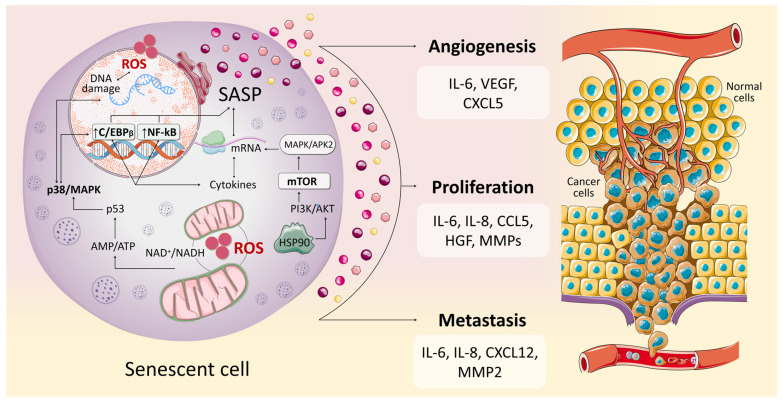
Key signaling pathways and mechanisms involved in the formation of the senescence-associated secretory phenotype (left side of the figure) and their impact on oncogenesis (right side of the figure). SASP is produced under the influence of various pathways, the key ones being NF-kB, PI3K-AKT-mTOR, p38 MAPK, and C/EBP-β, which are critically dependent on the production of reactive oxygen species. ROS and products formed as a result of reactions of the mitochondrial electron transport respiratory chain accumulate during cellular senescence and lead to the activation of the above pathways, and also themselves cause DNA damage. All this leads to increased secretion of SASP factors. In turn, SASP factors play a critical role in the pathogenesis of malignant neoplasms, affecting the tumor microenvironment. The chronic proinflammatory and immunosuppressive state formed under the influence of SASP increases the malignant potential of the cancer cell population. This is expressed in the strengthening of the processes of angiogenesis, proliferation, and metastasis. Abbreviations: IL-6, Interleukin-6; VEGF, Vascular endothelial growth factor; CXCL5, C-X-C motif chemokine 5; IL-8, Interleukin-8; CCL5, Chemokine (C-C motif) ligand 5; HGF, Hepatocyte growth factor; MMPs, Matrix metalloproteinases; CXCL12, C-X-C motif chemokine 12; MMP2, Matrix metalloproteinase-2.

The left part of Figure 1 presents generalized information about the pathways and mechanisms involved in SASP formation. Today there are a sufficient number of review studies published in authoritative journals to provide comprehensive information on the above-mentioned molecular mechanisms regulating SASP [34,82]. Consequently, in our manuscript we did not dwell on the activation of SASP genes by various transcription factors in order to avoid duplication of information already well-structured in other works. Instead, we focus our attention on the role of SASP specifically in the context of the development of malignant neoplasms.

It has now been convincingly demonstrated that senescent cells are a common feature of all malignant tissues. Thus, an increased number of senescent cells expressing the classical senescence factor—senescence-associated β-galactosidase (SA-β-gal)—correlates with an unfavorable prognosis in ovarian cancer [83,84], colorectal cancer, and other invasive colon tumors [85,86]. In turn, the aging marker CXCR2 (interleukin-8 receptor β), which has significant pro-tumor functions, is found everywhere in samples obtained from patients with colorectal [87], breast [88,89], stomach [90,91], pancreas [92] and esophagus [93] cancer; lung adenocarcinoma [94]; acute myeloid leukemia [95]; etc. A large number of studies have also demonstrated that changes in the expression of another biomarker of cellular senescence—the p16 protein—correlate with a poor prognosis in patients with oncology. In particular, this is observed in prostate cancer [96,97], anaplastic astrocytoma and glioblastoma [98], high-grade osteosarcoma [99], etc.

In addition to the intrinsic mechanisms of cell senescence, a significant number of antitumor interventions also induce aging in cancer cells [100]. As mentioned above, the induction of cellular senescence in response to therapeutic intervention leads to stable cell cycle arrest. This, it would seem, can be considered as a reliable independent strategy for the treatment of malignant neoplasms. Thus, in clinical practice today, the induction of aging is a key mechanism of many types of antitumor therapy, mainly radio- and chemotherapy. In particular, doxorubicin, one of the most widely prescribed first-line chemotherapeutic drugs, exerts a toxic effect through the formation of a state of stable proliferative arrest [101]. Similar effects have been shown for cisplatin, tamoxifen and a number of other drugs used in the clinic [17,20,102,103].

Nevertheless, despite the significant positive therapeutic effect of such drugs, the accumulation of senescent cells can cause long-term side effects and, most importantly, lead to the formation of the drug resistance phenomenon [104], as well as disease relapse [33]. It has been shown that SASP causes an acceleration in growth due to increased angiogenesis, migration, and metastasis (Figure 1) because of a change in the epithelial phenotype to mesenchymal in neighboring cancer cells [105,106]. In the work of Karabicici et al., the treatment of liver cancer cells with doxorubicin was accompanied by a significant increase in ABCG2 expression—the gene associated with multidrug resistance [107]. In turn, treatment of the HuH7 cell line with the drug led to an increase in the ability of the cell population to form tumors in vivo [108]. It has also been shown that 5-fluorouracil (5-FU)-induced cellular senescence in proliferating HCT 116 colorectal cancer cells affects the tumor microenvironment, promoting epithelial–mesenchymal transition and increased tumor invasiveness [109].

Moreover, it is known that the heterochromatinization of proliferative genes observed in senescent cells [110] leads to long-term rather than radical cell cycle arrest. This indicates the viability of senescent cells and the possible resumption of their pro-tumor functions. No less important is the fact that, once a certain number of senescent cells has been reached, the latter can have systemic toxic effects, causing various diseases associated with aging. In particular, there are direct correlations between the incidence of age-related diseases in patients receiving chemotherapy as a guideline-concordant form of cancer care. Thus, a recent study by Yuwono et al. [111] reported on a patient with stage IV colorectal cancer who received chemotherapy with high doses of 5-FU, who subsequently developed encephalopathy with stroke-like episodes. Also, the study presented by Florido et al. [112] convincingly proves a significantly higher risk of cardiovascular diseases (such as coronary heart disease, stroke, heart failure) among patients who have undergone therapy against cardiovascular diseases. This is also confirmed by a number of randomized clinical and large observational studies [113,114,115,116,117,118]. In turn, Tan et al. [119], in a meta-analysis, showed that the use of tamoxifen for the treatment of hormone-sensitive breast cancer may be associated with an increased risk of Parkinson’s disease in women. A similar correlation in the incidence of neurodegenerative disorder after tamoxifen therapy was found for patients with Alzheimer’s disease [120].

All this refutes the notion of a net positive effect of senescence on tumor growth suppression. There is no doubt that the persistence of senescent cells in oncological diseases makes a negative contribution to the therapy of malignant neoplasms. This indicates the prospects of using a strategy in cancer treatment protocols that achieves the elimination of senescent cells. In this way, it could minimize the risk of further tumor progression and avoid side effects. Despite the fact that selective eradication of senescent cells was originally developed for the treatment of age-related diseases, senolytic therapy is gaining momentum in the treatment of malignant neoplasms every year, and is also considered one of the main tasks in the fight against cancer [121].

## 3. Natural Products as Senotherapeutic Agents

**Figure 2:**
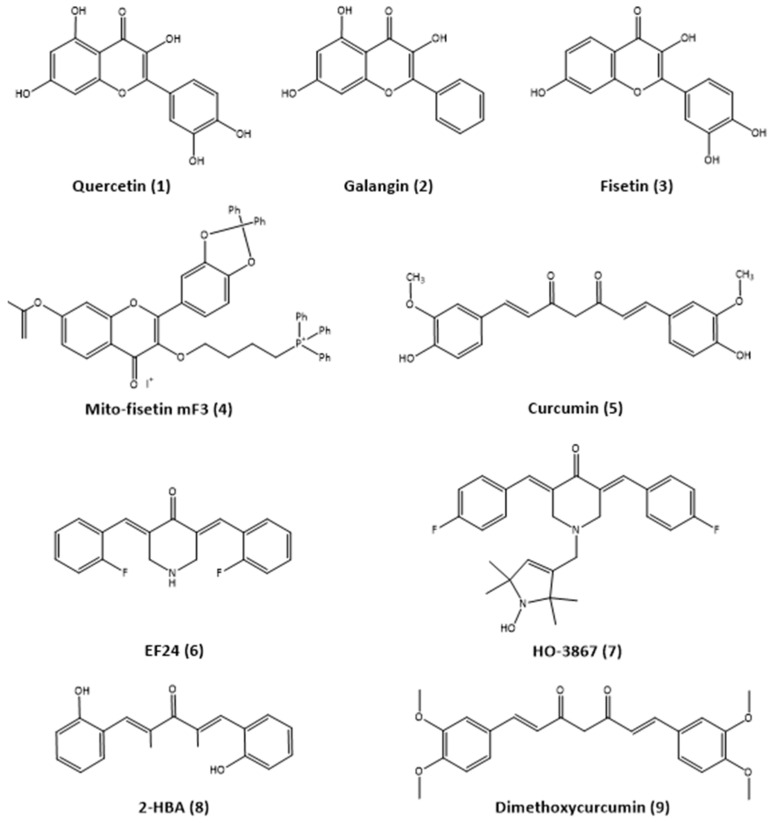
Structural formulas of natural molecules considered as potential therapeutic agents.

Natural products have been considered for centuries as invaluable sources of therapeutic agents of various pharmacological directions due to their wide structural diversity and inexhaustible spectrum of biological activity. Recently, interest in these molecular objects as senolytic agents has increased due to their low toxicity and the unlimited possibilities of their structural modification. In our review, we have focused on the natural compounds and their structural analogs shown in Figure 2. These molecules have a pronounced antioxidant potential, which can be considered as a useful tool for the eradication of senescent cells in oncotherapy.

### 3.1. Quercetin

Quercetin (3,5,7,3′,4′-pentahydroxyflavone, **1**) is a major member of the flavonoid subclass of flavonols, commonly found in many plant-based foods [122]. Quercetin is a potent antioxidant, due to the specific arrangement of the catechin and hydroxyl groups in the molecular structure. Quercetin has excellent ability to inhibit lipid peroxidation, neutralize free radicals, and bind metal ions [123] through the modulation of multiple intracellular signaling pathways that play a key role in carcinogenesis [124]. Moreover, in recent years, due to the encouraging results obtained by a number of research groups, the elimination of senescent cells by quercetin has become another attractive therapeutic strategy for cancer treatment.

As a well-known antioxidant that acts simultaneously on several nodes of the senescent cell anti-apoptotic pathway (SCAP) networks, quercetin was able to eliminate curcumin-induced senescent cancer cells HCT 116 [125]. A similar effect was also shown for a hydroxy-blocked quercetin derivative [126]. This molecule has the ability to sensitize etoposide-resistant senescent breast cancer cells to apoptotic death. This is due to the regulation of a pool of proinflammatory factors, as well as biomarkers of intracellular oxidative status p27 [127] and heat shock protein 90 (Hsp90) [128]. The antitumor effects of quercetin are also mediated by its effect on the expression of caspases, which are key mediators of apoptotic cell death [129,130]. In a colon cancer model, rats treated with quercetin at a dose of 10 mg/kg for 17 weeks exhibited a significant increase in caspase-3 expression. This was accompanied by a decrease in pathological cytological changes [131].

In addition to the well-documented adjuvant properties, Bientinesi et al. [132,133] found an equally unique cardioprotective effect of quercetin in a doxorubicin-induced model of cellular senescence. Thus, pretreatment with quercetin resulted in the alleviation of doxorubicin-induced cellular senescence in normal human WI-38 fibroblasts by reducing the number of senescent cells and the production of SASP factors. This also resulted in a reduction in the pro-tumor effects of the conditioned medium from doxo-induced senescent fibroblasts on osteosarcoma cells.

It is worth noting that the first registered senolytic agent was a cocktail consisting of quercetin and dasatinib. The therapeutic benefit of this mix has been proven in a number of experimental and clinical studies [26,134,135,136,137], and interest in the above-mentioned agent has only grown recently. In 2024 alone, when entering the phrase “quercetin dasatinib cancer” into the Pubmed search bar, there were 11 papers, including a study by Wang [138]. This work demonstrated the ability of quercetin + dasatinib to effectively restore glucose homeostasis in mice with ovarian cancer and significantly reduce the aging of adipose tissue, while the combination of the mixture with carboplatin or olaparib led to a noticeable decrease in the metastasis of ovarian cancer cells through the production of inflammatory cytokines and chemokines. At the same time, in the work of Ji et al., extracellular vesicles loaded with a senolytic combination effectively and selectively eliminated senescent cells in a syngeneic mouse model by stimulating antitumor immunity [139]. In addition, in vivo therapy with a senolytic cocktail led to the clearance of senescent cells after radiation therapy, thereby mitigating radiation ulcers [140], a common toxic side effect in patients receiving radiation therapy [141]. We suggest that the antioxidant properties of quercetin make a key contribution to the manifestation of this effect, since it is well known that cell aging during radiation therapy occurs as a result of intensification of oxidative stress and hyperproduction of ROS [142].

### 3.2. Galangin

Galangin (3,5,7-trihydroxyflavone, **2**) is a natural flavonoid found in high concentrations in *Alpinia officinarum* and other members of the ginger family and has been used in traditional Chinese medicine for many years. As a highly effective antioxidant, galangin exerts its therapeutic effects through a wide range of oxidative stress-related signaling pathways [143,144]. To date, there is no direct evidence in the literature for the senolytic action of galangin. However, there is a compelling body of evidence supporting the ability of the flavonoid to modulate the cellular senescence process. This is of interest in discussing its possible therapeutic effects as a potential senolytic.

In particular, in the study by Lee et al., it was found in vitro and in vivo that, due to the potent antioxidant activity mediated by the activation of the Nrf2/ARE signaling pathway, galangin reduces the level of H_2_O_2_/UV-induced intracellular and mitochondrial ROS production and, as a result, promotes the elimination of senescent cells [143]. Similar effects of galangin were shown in earlier studies on human keratinocytes subjected to UV-induced oxidative damage [145] and human dermal fibroblasts HS68 with a model of inflammation induced by H_2_O_2_ [146,147]. In the works of Song et al. [148] and Zhang et al. [149], the ability of galangin to exert a therapeutic effect through the activation of the AMPK signaling pathway was demonstrated. This was accompanied by the induction of autophagy and subsequent inhibition of proliferation and stimulation of apoptosis of tumor cells. It should be noted that the vital regulatory role of AMPK in cellular senescence has been convincingly proven [150]. Thus, it has been shown that, with age, a decrease in AMPK activity is observed in an aging organism [151]. In turn, the activation of AMPK leads to the blocking of this process and is capable of increasing the lifespan of organisms [152,153,154]. This is due to the intensification of autophagic capacity in relation to defective aging cells and the subsequent reduction in sluggish inflammation [155,156].

An interesting mechanism of antitumor action of the antioxidant senolytic galangin was demonstrated by Zhang et al. [157]. The team of authors discovered the ability of the flavonoid to increase the sensitivity of human esophageal cancer cells to the action of traditional chemotherapy with 5-FE. This synergistic effect of galangin is due to its inhibitory effect on the NLR family pyrin domain containing inflammasome 3 (NLRP3), a promising target of antioxidant action [158]. Similar effects of galangin have also demonstrated therapeutic benefit in in vivo studies on the cardiotoxicity of a combination of doxorubicin and galangin in mice [144]. In addition to blocking increases in IL-1β, IL-6, and TNF-α and, as a result, the inflammatory process, galangin reversed increases in the levels of ROS, malondialdehyde, and NADPH oxidase, as well as the suppression of superoxide dismutase activity via the Nrf2 signaling pathway. Apparently, this cardioprotective effect of the flavonoid may be due to the elimination of senescent cardiomyocytes induced by doxorubicin [159]. Interestingly, Huang et al. [160] demonstrated the ability of galangin to reduce cisplatin-induced nephrotoxicity by inhibiting the secretion of the proinflammatory factors IL-1β, TNF-α, and IL-6. Moreover, the ability of galangin to modulate p53 and Bax levels, as well as activate caspase-3, allowed the therapeutic effects of cisplatin to be enhanced. This underlines the prospects of considering galangin as a promising adjuvant agent.

### 3.3. Fisetin

Fisetin (3,3′,4′,7-tetrahydroxy-flavone, 5-deoxyquercetin, **3**) is secondary metabolite of most plants, found mainly in their green parts [161], and the first mentions of it date back to the 1830s. Currently, fisetin is of great interest due to its excellent antioxidant activity [162] and, as a result, its effect on numerous biological processes that play a key role in homeostasis. Thus, in the context of the implementation of antitumor effects, fisetin modulates cell viability by inhibiting proliferative activity [163], angiogenesis [164,165], and metastasis [163,166], as well as by inducing cell death through apoptosis [167,168].

There is information in the literature on the study of fisetin as a senolytic agent for the treatment of age-related diseases, but there are no reviews of these properties for the fight against malignant neoplasms. However, due to the specific nature of the review offered to the readers’ attention, we tried to analyze the antitumor properties of fisetin as a therapeutic agent that implements effects by eliminating senescent cells. It is interesting that in a number of studies, fisetin demonstrated the most powerful senolytic activity among such flavonoids as quercetin, resveratrol, curcumin, apigenin, etc. This is clearly seen in Table 2, showing a significantly more pronounced decrease in the number of SA-β-gal-positive mouse embryonic fibroblasts (MEFs) at a concentration of 5 μM [169].

In a double-blind, randomized, placebo-controlled clinical trial by Farsad-Naeimi et al. it was shown that daily use of 100 mg of fisetin in combination with chemotherapy with oxaliplatin and capecitabine for 7 weeks led to a significant decrease in the inflammatory markers IL-8, hs-CRP, and MMP-7 in the blood plasma of patients with colorectal cancer compared to the placebo group [170]. Such an effect of fisetin on proteins associated with aging certainly suggests significant antitumor effects, since it is well known that the cytokine IL-8, by potentiating cell aging [171,172], strengthens the tumorigenic capabilities of cancer cells, giving them more aggressive phenotypes [51]. No less important a role in the aging process and, as a consequence, the progression of malignant neoplasms, is played by C-reactive protein [173], realizing its effects through a Smad3-dependent p21/p27 mechanism [174]. Thus, fisetin improves the inflammatory status of patients with colorectal cancer, and further use of the therapy may reveal the positive effect of the flavonol as a senolytic agent.

The success of using fisetin in combination with known antitumor agents was confirmed in the work of Pal et al. [175]. The mix of the flavonoid with sorafenib made it possible to reduce the working concentration of the latter and achieve effective inhibition of human melanoma cell growth. This treatment resulted in increased apoptotic cell death. This increased expression of the proapoptotic proteins Bax and Bak and decreased levels of the antiapoptotic proteins Bcl2 and Mcl-1, as well as inhibiting PI3K protein expression and phosphorylation of AKT, MEK1/2, ERK1/2, and mTOR. Such modulation of some of the key regulators of cellular senescence [176] suggests that the observed effects of the fisetin + sorafenib combination in the in vitro model, as well as in athymic nude mice with implanted melanoma cells (A375 and SK-MEL-28) in vivo, may obviously be due to the senolytic potential of the former.

The ability to exert antitumor effects via the effect on apoptotic factors has also been demonstrated for fisetin itself. In particular, fisetin induced cell death in the human cervical carcinoma HeLa cell line via activation of caspases-3 and -8 [177]. This was also accompanied by a robust activation of ERK1/2 phosphorylation and significant antitumor effects in vivo in HeLa xenograft mice. The ability of fisetin to induce cancer cell death via caspase-dependent apoptosis has also been demonstrated in a recent study by Yadav et al. [178]. Moreover, oral treatment with fisetin at 50 mg/kg for 19 days in mice with head and neck squamous cell carcinoma xenograft model Cal33 resulted in significant inhibition of tumor mass along with an increase in cleaved caspase-3 levels and, consequently, a decrease in p21 and p27 protein levels.

In addition, fisetin conjugated with cationic triphenylphosphonium groups (mito-fisetin mF3, **4**) exhibited a much more potent antitumor effect in both the in vitro (against hormone-dependent breast cancer cell lines (HCC1500, CAMA-1, HCC1428, and ZR-75-30 cell lines) with tamoxifen-induced senescence) and the in vivo ER-positive breast cancer cell line-based xenograft system [179]. Thus, a significant decrease in the mitochondrial membrane potential and an intensification of oxidative stress were observed when using just 1 μM mitofisetin. This was accompanied by an increase in the level of phosphorylated AMPK, a decrease in the levels of AKT and Hsp90—markers of cellular senescence—[180,181], and, as a consequence, a violation of the mitophagic response. Apparently, conjugation of fisetin with cationic triphenylphosphonium groups allowed expansion of the senotherapeutic spectrum of the molecular action of the flavonoid by eliminating tumor cells due to dysfunction of mitophagy and enhancing apoptosis of senescent cells.

### 3.4. Curcumin

Curcumin (1,7-bis-(4-hydroxy-3-methoxyphenyl)-hepta-1,6-diene-3,5-dione, **5**) is a polyphenolic molecule isolated from the rhizome of *Curcuma longa* L., which has a broad spectrum of biological activity [182]. Curcumin has received its universal recognition mainly due to its pronounced antioxidant and anti-inflammatory [183] properties. This significantly contributes to the manifestation of its neuroprotective [184], antitumor [185], and antibacterial [186] potential.

In this review, we would like to pay special attention to the senotherapeutic potential of curcumin, whose synthetic analogs have been the subject of intensive research by the authors of this manuscript for several years [187,188,189]. The spectrum of the antitumor effects of curcumin and its derivatives discovered to date (in particular, antioxidant properties and effects on a number of targets potentially associated with cell senescence) is accompanied by a growing interest in direct study of the senolytic profile. Although the literature is rich in information on the opposite effect of curcumin, consisting in its ability to induce cell aging, in this review we will for the first time summarize the role of curcumin and its analogs in the utilization of senescent cells in the context of malignant neoplasm therapy.

As an antioxidant molecule, curcumin affects Nrf2, which is involved in the regulation of cellular senescence [190]. In the work of Yuan et al., curcumin reduced tumor volume in an osteosarcoma xenograft model by regulating the Nrf2/GPX4 signaling pathway [191]. Interestingly, the effects of the polyphenol were reversed by using inhibitors of each component of the signaling pathway. At the same time, a team of authors led by Liu managed to discover the ability of curcumin to activate the KEAP1/NRF2/ARE pathway in colorectal cancer cells. This was accompanied by apoptosis induction, suppression of migration and invasion, and the ability to enhance the therapeutic effects of 5-FE on tumor cells [192]. The use of curcumin as an adjuvant therapy to cisplatin in cells and in mouse models of ovarian cancer demonstrated an increase in Nrf2 expression. Moreover, a more promising profile of mitochondrial biogenesis markers, including PGC-1α and mitochondrial transcription factor A (TFAM) expression, has been identified, significantly increasing their levels. Importantly, stimulation of TFAM expression mitigated senescence, which has been demonstrated in a number of experimental studies [143,193].

In a study of the senolytic activity of curcumin analogs EF24 (3,5-bis (2-fluorobenzylidine)-4-pyperidone, **6**), HO-3867 (diarylidenylpieperidone (DAP)-inspired analog, (3E,5E)-3,5-bis[(4-fluorophenyl)methylidene]-1-[(1-hydroxy-2,2,5,5-tetramethylpyrrol-3-yl)methyl]piperidin-4-one **7**), 2-HBA ((1E,4E)-1,5-bis(2-hydroxyphenyl)-1,4-pentadien-3-one, **8**), and dimethoxycurcumin, their ability to reduce the survival of cells with ionizing radiation-induced senescence, in contrast to normal human fibroblasts WI-38, was discovered [194]. Interestingly, the most potent cytotoxic activity against senescent cells was detected for EF24 with an IC_50_ value of 1.62 μM and 4.69 μM on WI-38. It should be noted that the observed effect of EF24 was not specific only to the WI-38 cell line with a senescent phenotype induced by ionizing radiation. Similar effects of EF24 were observed on a panel of cells including human fibroblasts (IMR-90), human kidney epithelial cells (HREC), human umbilical vein endothelial cells (HUVEC), and human preadipocytes in a senescent state induced by various pathways. Interestingly, when studying the mechanisms of the cytotoxic effect of EF24, it was found that the compound induces apoptosis in senescent cells through a pathway independent of ROS production, significantly reducing the expression of Bcl-xl and Mcl-1 proteins. Mcl-1 and Bcl-xl have been identified as promising targets for senolytic cancer therapy [195,196,197,198,199,200]. In particular, degradation of Bcl-xl in hematopoietic cells and primary acute myeloid leukemia samples obtained from patients using PROTAC 753B was accompanied by an increase in the effectiveness of chemotherapy [201]. In a study by He et al. [202] on human colon cancer cell lines HCT-116, SW-620, and HT-29, it was shown that EF24 effectively induced cell death via the apoptotic pathway. This was accompanied by a decrease in Bcl-2 expression and an alteration in the Bcl-2/Bax ratio, as well as the induction of activation of both initiator caspase-9 and effector caspase-3. A similar effect on caspase activity and, as a consequence, the ability to lead to apoptotic events in cancer cells was also shown for HO-3867 [203]. HO-3867 effectively reduced the viability of the hepatocellular carcinoma cell line via the apoptotic pathway through p38-mediated activation of caspase cascades.

It is worth noting that curcumin itself also has this ability to suppress the overexpression of anti-apoptotic proteins associated with aging [204,205]. Interestingly, in [206], Yang and colleagues found that curcumin dose-dependently reversed senescence-associated β-galactosidase activity and overexpression of D-galactose-induced P53 and P16 proteins in cardiomyocytes. Similar effects of curcumin have been shown in dental follicle cells [207], porcine proximal tubule (LLC-PK1), and human kidney (HK-2) cell lines [208]. Also, a recent systematic review by Besasie et al. [209] concluded that one of the most common molecular and cellular pathways through which the natural molecule exerts its antitumor effects against prostate cancer are caspases-3 and -9, which is also supported by a number of other studies [210,211].

As mentioned above, high levels of matrix metalloproteinases are a critical marker of SASP. A considerable amount of data indicate the ability of both curcumin itself and its derivatives to inhibit the expression of these peptidases, which is accompanied by convincing antitumor effects. In particular, in the work of Sun et al. [212], treatment of endometrial cancer cells led to MMP-2 suppression, which led to the inhibition of cell invasion. An in silico study by Jerah et al. identified another protease isoform, MMP-3, as a potential target of polyphenol [213]. The potentiating effect of curcumin on capecitabine was found when using a combination therapy of cells and nude mice with metastatic colorectal cancer [214]. This was expressed in enhancing the antitumor and antimetastatic effects of the cytostatic by inhibiting the expression of NF-kB-regulated genes, including MMP-9. A similar effect on matrix metalloproteinase-9 in cells of another malignancy phenotype, human nasopharyngeal carcinoma, was found in the work of Su et al. [215] for the above-mentioned curcumin analog EF-24. Such transcriptional suppression of MMP-9 gene expression resulted in decreased invasiveness without affecting the viability of cancer cells treated with 12-O-tetradecanoylphorbol-13-acetate (TPA), a phorbol ester that promotes the spread of neoplastic cells [216]. The ability to inhibit invasion and metastasis through the regulation of metalloproteinase expression was also confirmed for curcumin in the work of Liao et al. [217]. In this study, curcumin demonstrated a concentration-dependent effect on the invasion rate of human lung adenocarcinoma A549 cells in a transwell assay. This correlated with a decrease in the MMP-22 level along with membrane-bound matrix metalloproteinase (MT1-MMP), which play an important role in the invasive and metastatic potential of cancer cells.

It is likely that the explanation for this ability of curcumin and its analogs lies in the antioxidant action of the molecules. There is evidence that reactive oxygen species are important mediators of metalloproteinases, increasing their expression [218]. Apparently, being powerful antioxidant molecules, curcumin and its derivatives have the ability to normalize the ROS content in tumor cells and thereby reduce the MMP expression. Thus, it is well known that cancer cells are characterized by a high level of oxidative stress. This assumption may find some confirmation in the recent work of Ibanez Gaspar et al. [219]. The 72 h treatment of human renal cell carcinoma (ACHN) with curcuminoids EF-24 and dimethoxycurcumin, as well as curcumin itself, led to suppression of intracellular H_2_O_2_ levels. This correlated with reduced activity of metalloproteinase isoforms 2 and 9 and inhibition of ACHN cell migration.

## 4. Conclusions

According to historical continuity, stimulation of senescence in response to oncogenic stimuli has been considered for many years as a protective mechanism against cancer, while “prosenescence” therapy of malignant neoplasms, aimed at inducing the formation of senescent cells, is a powerful antitumor strategy that suppresses the uncontrolled proliferation of neoplastic cells. Based on these claims, a large number of research groups have focused their attention on the development of new drugs and improvements to existing drugs that implement their antitumor effects through the TIS pathway. Some attempts to create such drugs have been successful and have proven their promise in preclinical and clinical trials, in particular, inhibitors of cyclin-dependent kinases, markers associated with senescence [220], Ribociclib [221,222,223], Abemaciclib [224,225,226], and Palbociclib [227,228].

However, over the years, this concept has lost its original meaning, and the contribution of senescent cells to the pathogenesis of malignant neoplasms has been underestimated. It has been found that the state of accumulation of senescent cells in cancer also has adverse consequences. These effects are associated with the maintenance of a viable state of cells forming a SASP, which in turn promotes local and systemic inflammation, as well as creating an immunosuppressive microenvironment.

Apparently, senescent cells have a “healing power” only in a controlled quantity and when their presence in the body is temporary. A simple example can serve as confirmation of this. It is well known that one of the positive functions of the aging process and the formation of SASP is the ability to effectively restore damaged tissues. In particular, a significantly higher degree of fibrosis was observed in injured genetically modified mice with a deficiency of the p53/p21 and p16INK4a/pRB pathways, i.e., dysfunction of the aging process [229]. Exacerbation of fibrosis and, as a result, disturbances in the healing of skin wounds were also observed in wild-type mice expressing the mutant matricellular protein CCN1 [230], which is a marker of cellular senescence [231,232,233]. Furthermore, the cellular senescence induced by this protein led to increased regeneration of the hearts of newborn mice, mediated by a decrease in cardiac tissue fibrosis and increased proliferation of cardiomyocytes [234]. All this confirms the hypothesis of the healing role of senescence and aging-related factors. In turn, it is paradoxical that the increase in the number of senescent cells with age does not correlate at all with an improvement in the rate of tissue recovery and healing. On the contrary, in older people, wound healing is seriously slowed down and is accompanied by other negative consequences [235]. Apparently, this can be explained by the fact that the above-mentioned examples of the therapeutic benefits of cellular senescence imply an episodic presence of senescent cells and their subsequent senolysis by the immune system. In turn, the chronic presence of senescent cells in the body is accompanied by a number of negative consequences.

**Figure 3:**
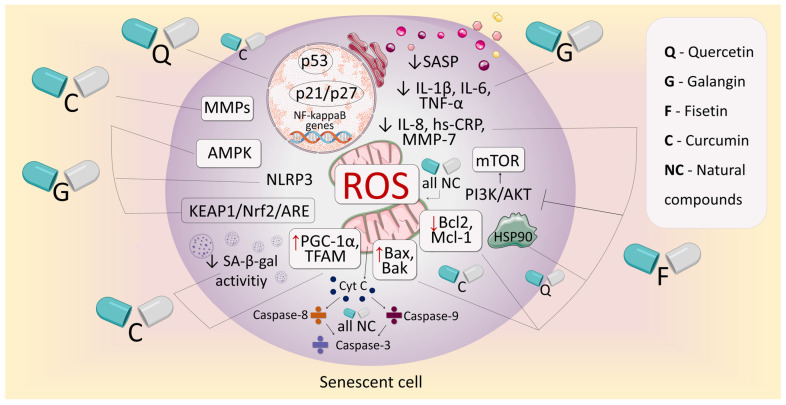
A generalized image of the senotherapeutic potential of the reviewed natural products with pronounced antioxidant activity. Abbreviations: ROS, Reactive oxygen species; SASP, Senescence-associated secretory phenotype; IL-1β, Interleukin-1β; IL-6, Interleukin-6; IL-8, Interleukin-8; MMPs, Matrix metalloproteinases; MMP-7, Matrix metalloproteinase 7; TNF-α, Tumor necrosis factor α; hs-CRP, High-sensitivity C-reactive protein; mTOR, Mammalian target of rapamycin; PI3K, Phosphatidylinositol 3-kinase; Bak, Apoptosis regulator Bak; Bax, Apoptosis regulator Bax; Bcl2, Apoptosis regulator Bcl-2; Hsp90, Heat shock protein 90; Mcl-1, Induced myeloid leukemia cell differentiation protein; PGC-1α, Peroxisome proliferator-activated receptor gamma coactivator 1-α; TFAM, Mitochondrial transcription factor A; SA-β-gal, Senescence-associated β-galactosidase; Nrf2, Nuclear factor erythroid 2-related factor 2; NLRP3, NLR family pyrin domain containing 3 inflammasome; AMPK, AMP-activated protein kinase; ARE, antioxidant response element. The down arrow indicates an inhibition of the activity or expression of factors; the up arrow indicates an increase in the expression of factors.

Thus, it becomes obvious that the well-proven “prosenescence” cancer therapy should not be included in the protocols for the treatment of malignant neoplasms as an independent unit. This is due to the increased viability of senescent cells and reversibility of cell cycle arrest, as well as the formation of a state of chronic inflammation and immune dysregulation, which ensures the manifestation of side effects. All these problems emphasize the need to search for senolytic agents that can be used as an adjuvant therapy in sequential cancer treatment regimens, improving the prognosis in patients after therapeutic intervention. In our review, we have demonstrated that one of the promising approaches to the creation of such drugs may be the use of natural products with a pronounced antioxidant potential. This potential is due to the participation of individual components of redox signaling pathways in cellular senescence. In Figure 3, we present generalized information about the mechanisms of the senolytic action of such molecules.

Although the use of natural molecules as senolytics is not currently widespread in clinical practice due to their less pronounced therapeutic activity than that of synthetic therapeutic agents of targeted action, the absence of serious side effects gives natural products a great chance of being used as leading tools against aging cells. Moreover, it has become evident that a serious constraint on the use of such molecules as therapeutic agents in vivo is the limited pharmacokinetic characteristics of natural compounds. Despite the wide range of biological properties exhibited by phytochemicals, the clinical efficacy of native molecules is weak. For example, the excellent antitumor effects of curcumin in in vitro models of prostate cancer [236,237,238] did not correlate positively with the therapy of patients with metastatic castration-resistant prostate cancer in combination with docetaxel [239]. This is explained by the low bioavailability and rapid metabolism of the polyphenol [240], which leads to the need to discontinue such clinical trials. Similar obstacles stand in the way of introducing other natural molecules into clinical practice. It is no secret that in this case the key to success is the development of a finished dosage form that would surpass the pharmacokinetic parameters of native phytomolecules. In this regard, a relevant direction is the improvement of the pharmacokinetic characteristics of natural compounds by obtaining synthetic analogs based on them [241] or using targeted delivery systems [242,243,244]. Overcoming the limitations associated with low oral bioavailability and rapid metabolism of natural products will certainly pave the way to the full use of the therapeutic possibilities of phytomolecules.

## Figures and Tables

**Table 1:** Factors whose secretion varies significantly in senescent cells.

SASP Factor	Features of Secretion in Senescent Cells
Soluble factors	
Matrix metalloproteinases (MMPs)	- Increased expression of MMP-1 [35], MMP-3 [35,36], MMP-9 [37], MMP-10 [36], MMP-12 [38], MMP-13 [39] - Decreased expression of MMP-8 [40]
Tissue inhibitors of metalloproteinases (TIMPs)	- Deficiency of TIMP1 [41], TIMP2 [42]
Plasminogen activator inhibitors (PAIs)	- Increased expression of PAI1 [43,44]
Cathepsins (CTSs)	- Increased expression of CTS B [45], CTS L [46] и CTS S [47] - Decreased level of CTS D [48]
Interleukins (ILs)	- Increasing levels of IL-1α [49], IL-1β [50], IL-6 [51,52], IL-8 [51], IL-10 [53], IL-13 [54]
Growth factors	- Increased secretion of amphiregulin (AREG) [55,56], epiregulin (EREG) [57], angiogenin [58], insulin-like growth factor binding proteins (IGFBPs) -3 [59], -4 [60], -5 [61] - Decreased expression of vascular endothelial growth factor (VEGF) [62,63], placental growth factor (PlGF) [64], beta-nerve growth factor (NGF) [65], IGFBP -2 [66], -6 [67]
Chemokines	- Increased concentration of monocyte chemoattractant proteins (MCPs) -1 [68], -2 [69], -3 [70], growth-regulated protein alpha (GRO α) [71], eotaxin-1 (CCL11) [72,73]
Non-protein factors	- Increased production of reactive oxygen species (ROS) [74]; reactive nitrogen species (RNS) [74]
Insoluble factors	
Glycoproteins	- Increased expression of fibronectin [75], vitronectin [76], laminin [77] - Depletion of collagen [78]

**Table 2:** Features of senolytic activity of flavonoids.

Natural Product	Senolytic Effects
Decreased number of SA-β-gal-positive MEFs	
Resveratrol	<10%
Fisetin	>50% *
Luteolin	>20%
Rutin	<10%
Epigallocatechin gallate	<10%
Curcumin	>30% *
Pirfenidone	<10%
Apigenin	<10%
Myricetin	<10%
Catechin	<10%
Quercetin	<10%

* *p* < 0.05 (two-tailed unpaired Student’s *t*-test).

## Data Availability

Not applicable.

## Abbreviations

The following abbreviations are used in this manuscript:

ABCG2	ATP binding cassette subfamily G member 2
AMPK	AMP-activated protein kinase
ARE	antioxidant response element
AREG	Amphiregulin
Bak	Apoptosis regulator Bak
Bax	Apoptosis regulator Bax
Bcl2	Apoptosis regulator Bcl-2
C/EBP-β	CCAAT enhancer binding protein beta
CCL5	Chemokine (C-C motif) ligand 5
CCL11	Eotaxin-1
CCN1	CCN family member 1
CXCL5	C-X-C motif chemokine 5
CXCL12	C-X-C motif chemokine 12
CTSs	Cathepsins
CXCR2	Interleukin-8 receptor β
DNA	Deoxyribonucleic acid
EREG	Epiregulin
GRO α	Growth-regulated protein α
H2O2	Hydrogen peroxide
HGF	Hepatocyte growth factor
hs-CRP	High-sensitivity C-reactive protein
Hsp90	Heat shock protein 90
IGFBPs	Insulin-like growth factor binding proteins
ILs	Interleukins
MAPK	Mitogen-activated protein kinase
Mcl-1	Induced myeloid leukemia cell differentiation protein
MCPs	Monocyte chemoattractant proteins
MMPs	Matrix metalloproteinases
mTOR	Mammalian target of rapamycin
NADPH	Nicotinamide adenine dinucleotide phosphate
NF-κB	Nuclear factor NF-kappaB pathway
NGF	β-nerve growth factor
NLRP3	NLR family pyrin domain containing 3 inflammasome
Nrf2	Nuclear factor erythroid 2-related factor 2
PAIs	Plasminogen activator inhibitors
PGC-1α	Peroxisome proliferator-activated receptor gamma coactivator 1-α
PI3K	Phosphatidylinositol 3-kinase
PlGF	Placental growth factor
RNS	Reactive nitrogen species
ROS	Reactive oxygen species
SASP	Senescence-associated secretory phenotype
SA-β-gal	Senescence-associated β-galactosidase
SCAP	Senescent cell anti-apoptotic pathway
SIPS	Stress-induced premature senescence
SMS	Senescence-messaging secretome
TFAM	Mitochondrial transcription factor A
TIMPs	Tissue inhibitors of metalloproteinases
TIS	Therapy-induced senescence
TNF-α	Tumor necrosis factor α
TPA	12-O-tetradecanoylphorbol-13-acetate
UV	Ultraviolet
VEGF	Vascular endothelial growth factor
**Cell lines**	
A375	Human melanoma
ACHN	Human renal adenocarcinoma
Cal33	Human tongue squamous cell carcinoma
CAMA-1	Luminal-type human breast cancer
HCC1428	Adenocarcinoma of the human mammary gland
HCC1500	Ductal carcinoma of the human mammary gland
HCT 116	Human colon cancer
HeLa	Human cervical adenocarcinoma
HK-2	Human kidney epithelial cell line
HREC	Human retinal microvascular endothelial cell line
HS68	Human fibroblasts from the foreskin
HuH7	Human hepatocarcinoma
HUVEC	Human umbilical vascular endothelial cells
IMR-90	Human lung fibroblasts
LLC-PK1	Pig kidney-derived cell line
MEFs	Mouse embryonic fibroblasts
SK-MEL-28	Human melanoma
WI-38	Human fibroblasts from the lung tissue
ZR-75-30	Infiltrating ductal carcinoma of the human mammary gland
